# Healthcare provider and drug dispenser knowledge and adherence to guidelines for the case management of malaria in pregnancy in the context of multiple first-line artemisinin-based combination therapy in western Kenya

**DOI:** 10.1186/s12936-023-04692-2

**Published:** 2023-09-08

**Authors:** Caroline B. Osoro, Stephanie Dellicour, Eleanor Ochodo, Taryn Young, Feiko ter Kuile, Julie R. Gutman, Jenny Hill

**Affiliations:** 1 Centre for Global Health Research, Kenya Medical Research Institute, P.O. Box 1578, Kisumu, 40100 Kenya; 2 Division of Epidemiology and Biostatistics, Department of Global Health, Faculty of Medicine and Health Sciences, Stellenbosch University, P.O. Box 241, Cape Town, 8000 South Africa; 3 Department of Clinical Sciences, Liverpool School of Tropical Medicine, Liverpool Pembroke Place, Liverpool, L3 5QA UK; 4 Malaria Branch, Division of Parasitic Diseases and Malaria, U.S. Centers for Disease Control and Prevention, 1600 Clifton Road, Atlanta, GA 30333 USA

**Keywords:** Anti-malarials, Artemisinin-based combination therapy, Malaria, Pregnancy, Case management, Healthcare providers, Knowledge, Health practices, Kenya

## Abstract

**Background:**

Concerns about emerging resistance to artemether-lumefantrine (AL) in Africa prompted the pilot introduction of multiple first-line therapies (MFT) in Western Kenya, potentially exposing women-of-childbearing-age (WOCBA) to anti-malarials with unknown safety profiles in the first trimester. The study assessed healthcare provider knowledge and adherence to national guidelines for managing malaria in pregnancy in the context of the MFT pilot.

**Methods:**

From March to April 2022, a cross-sectional study was conducted in 50 health facilities (HF) and 40 drug outlets (DO) using structured questionnaires to assess pregnancy detection, malaria diagnosis, and treatment choices by trimester. Differences between HF and DO providers and between MFT and non-MFT HFs were assessed using Chi-square tests.

**Results:**

Of 174 providers (77% HF, 23% DO), 56% were from MFT pilot facilities. Most providers had tertiary education; 5% HF and 20% DO had only primary or secondary education. More HF than DO providers had knowledge of malaria treatment guidelines (62% vs. 40%, p = 0.023), received training in malaria in pregnancy (49% vs. 20%, p = 0.002), and reported assessing for pregnancy in WOCBA (98% vs. 78%, p < 0.001). Most providers insisted on parasitological diagnosis, with 59% HF using microscopy and 85% DO using rapid diagnostic tests. More HF than DO providers could correctly name the drugs for treating uncomplicated malaria in the first trimester (oral quinine, or AL if quinine is unavailable) (90% vs. 58%, p < 0.001), second and third trimesters (artemisinin-based combination therapy) (84% vs. 70%, p = 0.07), and for severe malaria (parenteral artesunate/artemether) (94% vs. 60%, p < 0.001). Among HF providers, those in the MFT pilot had more knowledge of malaria treatment guidelines (67% vs. 49%, p = 0.08) and had received training on treatment of malaria in pregnancy (56% vs. 32%, p = 0.03). Few providers (10% HF and 12% DO) had adequate knowledge of malaria treatment in pregnancy, defined as the correct drug and dose for uncomplicated and severe malaria in all trimesters.

**Conclusions:**

Knowledge of national malaria in pregnancy treatment guidelines among providers in Western Kenya is suboptimal. Robust training on appropriate anti-malarial and dosage is needed, particularly given the recent change in recommendation for artemether-lumefantrine use in the first trimester. Supervision of DO and HF practices is essential for correct treatment of malaria in pregnancy in the context of MFT programmes.

**Supplementary Information:**

The online version contains supplementary material available at 10.1186/s12936-023-04692-2.

## Background

Malaria remains a significant public health challenge and a major cause of morbidity and mortality. In 2021, 247 million cases were reported, with 89% of all pregnancies in the World Health Organization Africa region (WHO AFRO) at risk of infection [1, 2]. Until November 2022, the WHO recommended oral quinine (first trimester) and artemisinin-based combination therapy (ACT) (second and third trimesters) for uncomplicated malaria in pregnancy and parenteral artesunate for severe malaria (all trimesters) [3, 4]. ACT was not recommended in the first trimester because of a lack of safety data in early pregnancy. Following a review of the safety evidence in 2022, the WHO now also recommends artemether-lumefantrine (AL) for treating uncomplicated malaria in the first trimester [5, 6].

Studies in several sub-Saharan African countries have shown that private health facilities and drug outlet providers lack knowledge and do not adhere to national malaria case management guidelines [7–12]. This was confirmed by recent meta-analyses that reported healthcare provider reliance on clinical malaria diagnosis and low adherence to appropriate malaria case management guidelines [13–15]. In Kenya, gaps in knowledge have been reported, with drug outlet providers, in particular, showing a poor understanding of national malaria treatment guidelines and a lack of training in malaria case management [16, 17]. A 2020 study of hospitals in Western Kenya’s Lake Victoria malaria-endemic region reported reliance on clinical malaria diagnosis even with the availability of rapid diagnostic tests (RDTs) [18], despite the national guideline that all suspected malaria cases be diagnosed by either microscopy or RDT [19]. A 2013 study in the same area reported correct knowledge of case management of malaria in pregnancy among healthcare providers in 45% of health facilities compared to 0% in drug outlets [20]. Furthermore, prescription of the correct drug and dosage according to trimester was practiced in only 62% of health facilities and 42% of drug outlets [20].

In response to the emerging threat of *Plasmodium falciparum* partial resistance to artemisinin-based combinations [21–26] and the consequent WHO recommendation to diversify ACT [27], in 2020, the Kenyan government embarked on a pilot study on the feasibility of rotational multiple first-line therapies (MFT) for malaria in the lake malaria-endemic region. The pilot includes rotation between AL, pyronaridine-artesunate (PA), dihydroartemisinin-piperaquine, and amodiaquine-artesunate (Pers. commun, Kokwaro, 2023). The introduction of MFT is likely to increase the exposure of women in early pregnancy to the newer generation of artemisinin-based combinations, such as PA, that have not yet been recommended for use in the first trimester. This is because women of childbearing age (WOCBA) are not routinely screened for pregnancy and either do not know or report they are pregnant and are, therefore, treated with the same drugs as used in non-pregnant adults. In 2021, a prospective, observational study (MiMBa pregnancy registry) was launched in Homa Bay to generate more evidence on the safety of the ACT used in the MFT pilot (Clinical trials registration NCT04825782).

The study sought to assess knowledge and adherence to malaria diagnosis and treatment guidelines by trimester among healthcare providers treating WOCBA in the context of the Kenyan Ministry of Health MFT pilot in Western Kenya.

## Methods

### Study design

A cross-sectional survey was conducted among healthcare providers working in health facilities and drug outlets to determine their knowledge of guidelines for the case management of malaria in pregnancy and factors associated with correct practice. The survey was part of a mixed methods study; the qualitative component is reported elsewhere (Pers. commun, Osoro, 2023). The survey has been reported following the Strengthening the Reporting of Observational studies in Epidemiology (STROBE) statement [28].

### Study site and sampling

The study was conducted from March to April 2022 in Homa Bay County in Western Kenya, along the shores of Lake Victoria. Malaria transmission is perennial and holo-endemic, with peaks following the two rainy seasons, March through May and October through December. In collaboration with private partners, the government piloted rotational multiple first-line treatments for malaria (MFT) in all county government and private not-for-profit health facilities (excluding private facilities and drug outlets) between June 2020 and September 2022. Training of healthcare providers on malaria case management was done as part of the roll-out of the MFT project. Between June 2020 and March 2021, AL was used as first-line treatment for uncomplicated malaria in adults, pregnant women in the second and third trimesters and children aged over 24 months in all sites. Between April and November 2021, AL was replaced by dihydroartemisinin-piperaquine as the first-line treatment on the mainland and by pyronaridine-artesunate on Mfangano and Rusinga Islands. The recommendation was to continue treating pregnant women according to the national treatment guidelines (seven days of oral quinine or AL if quinine is unavailable in the first trimester, AL in the second and third trimesters). The MiMBa Pregnancy Registry study operated in 35 public and private health facilities in the MFT pilot area. No training on case management was provided as part of MiMBa as this was an observational study.

### Sample size

Sample sizes were calculated using two-sided 95% confidence intervals for estimating a single proportion from a finite population (PASS 14, NCSS, LLC. Kaysville, Utah). Assuming 70% of providers had adequate knowledge and prescribing practice, a sample size of 50 health facilities from a total of around 70 was needed to obtain 95% confidence intervals (CI) equal to the sample proportion plus or minus 0.068 (63–77%). For the drug outlets, a sample size of 40 from an estimated total of 200 was needed to produce a two-sided 95% confidence interval equal to the sample proportion plus or minus 0.127 (57–83%).

### Participants

**Fig. 1:**
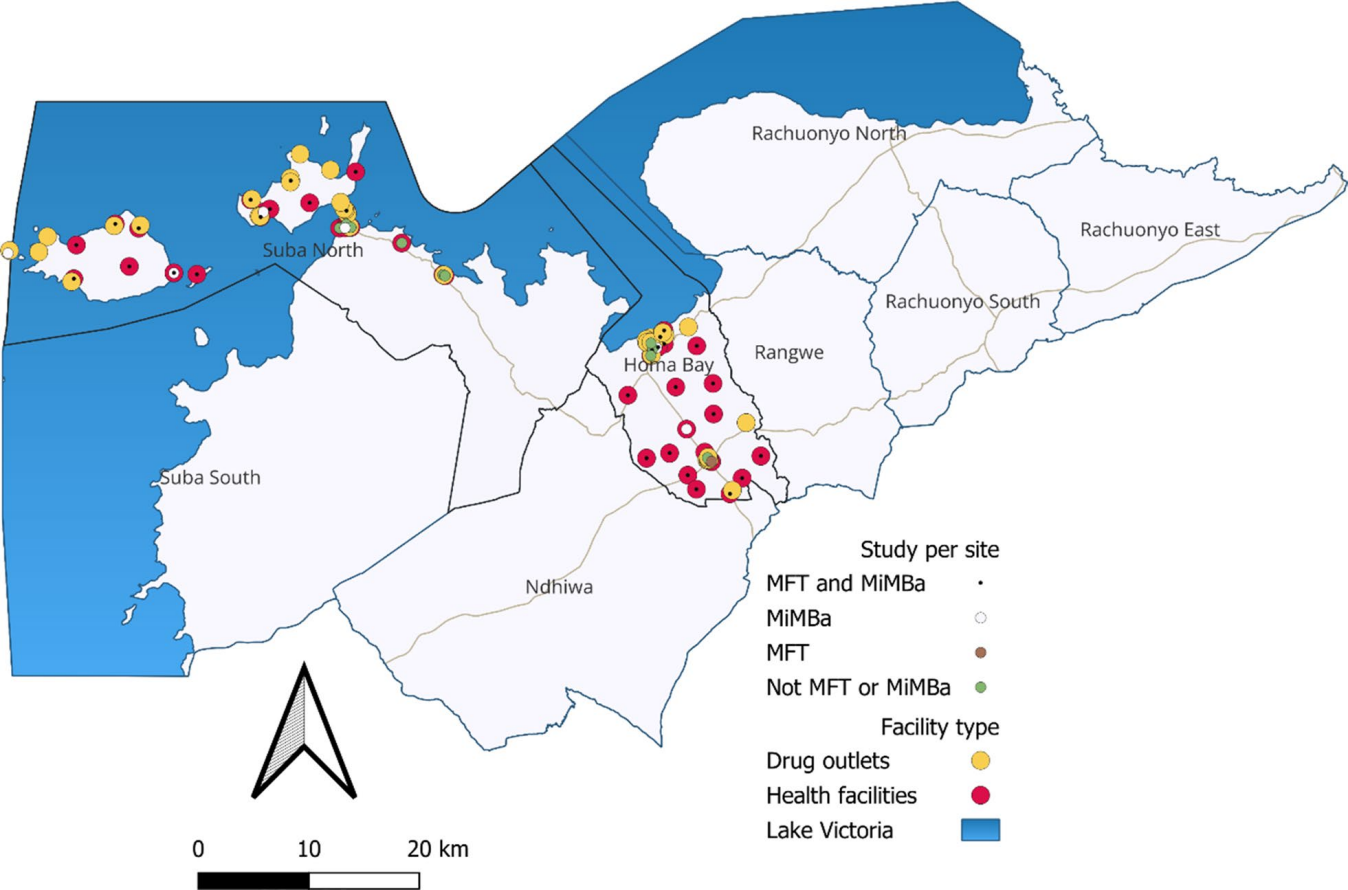
Map showing health facility and drug outlet distribution in Homa Bay county (MFT- Multiple first-line therapies)

The study population consisted of purposively selected healthcare providers responsible for treating adult outpatients, including pregnant women, or working at the pharmacy of health facilities. One person who was available during the study team’s visit was interviewed in drug outlets. For each health facility, at least one healthcare provider responsible for seeing adult outpatients or working at the pharmacy was interviewed (range 1–5). A total of 50 health facilities and 40 drug outlets were included. To be included, health facilities and drug outlets had to be operational and have anti-malarials stocked at any time in the last three months. Providers who did not fit the inclusion criteria and did not consent to participate were excluded. All 35 health facilities involved in the MiMBa study at the time were included; 33 of these were also participating in the MFT pilot. To meet the sample size requirements, an additional 15 health facilities and 40 drug outlets were randomly selected from a list of 57 facilities and 50 drug outlets selling anti-malarials compiled by community health volunteers (CHVs). Drug outlets were privately owned and not involved in either project (Fig. 1).

### Data collection procedures

A structured questionnaire was developed in English and pretested with ten participants with similar characteristics to the final study participants. Interviews were conducted face-to-face, lasting approximately 30–40 min, and data were collected using personal digital assistant devices. Respondents did not receive compensation for their time.

### Variables and measurements

The structured questionnaire was developed based on tools from a previous study [20] and considering the context of MFT and MiMBa projects ongoing at the time. The collected data included the sociodemographic characteristics of healthcare providers, including age, gender, education, professional qualification, job cadre and length of service. Data on awareness of the national malaria treatment guidelines, access to a copy of the treatment guidelines, presence of a job aid, on-job training in malaria in pregnancy, and initiative by the government to disseminate guidelines were collected. Information on facility characteristics was collected, including the type of facility, the managing authority, and participation in the MFT and MiMBa projects. Information on current practice in the case management of malaria in pregnancy was collected, including assessing for pregnancy in women of childbearing age, malaria diagnosis, presumptive clinical malaria treatment, and treatment for uncomplicated and severe malaria in different trimesters. Following data cleaning, recoding for one variable (pregnancy assessment) was done. Healthcare providers who answered ‘often’ or ‘sometimes’ for pregnancy assessment were grouped and compared to those who answered ‘never’. A knowledge variable was created based on the responses for the correct drug and dosage used to treat uncomplicated and severe malaria in different trimesters.

### Definitions

Adequate knowledge definitions were based on the 2020 Kenya national guidelines for diagnosing and treating malaria and the 2021 WHO Malaria treatment guidelines [3, 19] (Table 1). Healthcare providers were considered to have adequate treatment knowledge if they could correctly name the nationally recommended drug and dose (either the actual milligrams dosage or the number of tablets) for each trimester for uncomplicated and severe malaria.

**Table 1:** Definitions of correct practice and adequate knowledge for malaria case management in pregnancy

Correct Malaria diagnosis Parasitological confirmation/test by microscopy or rapid diagnostic test Clinical diagnosis when a test is unavailable
Correct pregnancy assessment Asked about last menstrual period, gestational age, and/or palpated for fundal height Asked about pregnancy and/or offered a pregnancy detection test
Correct treatment and dosage
Acceptable knowledge answers:
*First trimester- uncomplicated malaria*
Oral quinine and clindamycin. Artemether-lumefantrine if quinine is unavailable
*Second/Third trimester- uncomplicated malaria*
Oral artemether-lumefantrine or quinine and clindamycin
*Complicated/severe malaria (first, second and third trimesters)*
Parenteral artesunate or artemether followed by artemether-lumefantrine
Parenteral quinine followed by artemether-lumefantrine or oral quinine
*Treatment regimens and dosage*
Quinine: 2 tablets of 300 mg, 3 times daily for 7 days (2 × 3 × 7) and Clindamycin 150 mg twice daily for 7 days
Artemether-lumefantrine tablets (20/120 mg): 4 tablets, 2 times daily for 3 days (4 × 2 × 3)
Parenteral artesunate: Loading dose of 2.4 mg/kg body weight, then at 12 h and at 24 h, then once a day until the patient can tolerate oral medication
Parenteral artemether: Intramuscular 3.2 mg/kg, then 1.6 mg/kg every 24 h until the patient can tolerate oral medication
Parenteral quinine: Intravenous infusion 20 mg/kg body weight loading dose in 15mls/kg of 5% dextrose or normal saline, then 10 mg/kg every 8 h until the patient can tolerate oral medication

### Data management and analysis

Healthcare providers were categorized by facility type (health facility or drug outlet) and by participation in the MFT or MiMBa projects. Descriptive analyses were undertaken using frequencies and percentages. Chi-square tests were used to test differences in proportions; Fisher’s exact test was used where groups had five or fewer observations. Logistic regression models at the healthcare provider level were used to determine factors associated with correct case management. Healthcare provider variables examined were age, sex, respondent cadre, education, facility type, participation in the MFT or MiMBa project and in-service training on case management of malaria in pregnancy. Results from multivariable models are presented as adjusted odds ratios (aOR) with p-values and 95% CI at a significance level of 0.05. All analyses were conducted using R version 4.1.3.

### Ethical considerations

The study was approved by the Kenya Medical Research Institute’s Scientific and Ethics Review Unit (4277), the Stellenbosch University Health Research Ethics Committee (S21/03/056), and the Liverpool School of Tropical Medicine Research Ethics Committee (21–049). This study was reviewed by CDC and was conducted consistent with applicable US federal law and CDC policy. Participants provided written informed consent.

### Patient and public involvement

Community health volunteers (CHVs) living in the study area were engaged and remunerated to develop the list of all health facilities and drug outlets that sold anti-malarials. CHVs are lay members of the community who work either for pay or as volunteers in association with the local healthcare system in urban and rural environments. CHVs share ethnicity, language, socioeconomic status, and life experiences with the community members they serve and typically serve where they live [29].

## Results

### Characteristics of healthcare providers

A total of 174 healthcare providers were surveyed across 50 health facilities and 40 drug outlets; 56% (97/174) were male, and the mean age was 33 years (SD 7.6). The majority (77%, 134/174) were from health facilities, while 23% (40/174) were from drug outlets. Approximately half (53%, 92/174) were from private facilities, 41% (72/174) were from government facilities, and 6% (10/174) were from faith-based facilities. Amongst the health facility respondents, 52% (70/134) were from a health centre, 34% (45/134) were from a dispensary, and 14% (19/134) were from a hospital. Among drug outlet providers, 88% (35/40) were from a registered pharmacy, and 12% (5/40) were from a non-pharmacy drug store. The respondents comprised 36% (63/174) nurses, 30% (52/174) clinical officers, 26% (45/174) pharmacists or pharmaceutical technicians, and 8% (14/174) other (community health volunteers, teacher, businessperson). Most providers had received higher education, but a few had completed only primary or secondary education, 5% (6/134) from health facilities and 20% (8/40) from drug outlets. A relatively large proportion- 40% (54/134) of health facility providers and 30% (12/40) of drug outlet providers- had spent a year or less in their current position. Nearly three-quarters of the health facility respondents (72%, 97/134) were from the MFT project and MiMBa study facilities (Table 2).

**Table 2:** Healthcare provider/drug dispenser characteristics by facility type

	Overall		Health facilities		Drug outlets	
Characteristics	N = 174	%	N = 134	%	N = 40	%
Age in years- Mean (SD)	32.9 (7.6)		32.7 (7.0)		33.6 (9.6)	
Sex (Male)	97	56	70	52	27	68
Length of service in the current position						
A year or less	66	38	54	40	12	30
More than a year and up to five years	86	49	67	50	19	48
More than five and up to ten years	14	8	8	6	6	15
More than 10 years	8	5	5	4	3	7
Respondent cadre						
Clinical Officer	52	30	49	37	3	8
Enrolled Nurse	7	4	6	4	1	2
Registered Nurse	56	32	52	39	4	10
Pharmacist/Pharmaceutical Technician	45	26	21	16	24	60
CHV/other*	14	8	6	4	8	20
Level of Education/Certification						
Primary School	1	1	0	0	1	2
Secondary School	13	8	6	5	7	18
Tertiary School/ higher education	160	92	128	96	32	80
Clinical Officer	49	28	46	34	3	8
Registered Midwife/Nurse (3-year diploma)	53	30	50	37	3	7
Enrolled Midwife/Nurse (2-year certificate)	9	5	8	6	1	2
Pharmacist	11	6	5	4	6	15
Other*	38	22	19	14	19	48
Facility type						
Hospital	19	11	19	14	–	–
Health center	70	40	70	52	–	–
Dispensary	45	26	44	34	–	–
Registered pharmacy	35	20	–	–	35	88
Non-pharmacy drug store	5	3	–	–	5	12
Facility managing authority						
Government	72	41	72	54	–	–
Mission	10	6	10	7	–	–
Private	92	53	52	39	40	100
Participating in MFT** project and/or MiMBa study	97	56	97	72	0	0

*CHV- community health volunteer, ‘other’ included peer mentor mother, teacher, businessperson, receptionist, laboratory technologists.

**MFT- Multiple first-line therapies

### Knowledge of national malaria treatment guidelines

Over half of health facility providers (62%, 83/134) were aware of the existence of national malaria treatment guidelines, compared to 40% (16/40) of drug outlet providers (p = 0.023) (Table 3). Awareness of guidelines was higher among providers involved in the MFT and MiMBa projects (67% vs. 49%, p = 0.08) (Table 4). Most providers from health facilities compared to drug outlets (58% vs. 25%) had a job aid for malaria case management. Very few drug outlets compared to health facility providers (7% vs. 64%) reported being a recipient of any government initiative that disseminated guidelines (Table 5). Whereas 49% (66/134) of health facility providers received training in malaria in pregnancy, only 20% (8/40) of drug outlet providers had been trained (p = 0.002) (Table 3). Among health facility providers, more from the MFT and MiMBa sites than those not involved (56% vs. 32%, p = 0.03) had been trained on malaria in pregnancy (Table 4). Most health facility providers (62%) and those from MFT sites (69%) had been trained in the year preceding the survey (Table 5).

**Table 3:** Provider knowledge of national malaria treatment guidelines in pregnancy by facility type

	Overall			Health facilities			Drug outlets			p-value*
	n = 174	%	95% CI	n = 134	%	95% CI	n = 40	%	95% CI	
Awareness of MTGs	99	57	(49–64)	83	62	(52–70)	16	40	(26–55)	0.023
Training in MiP	74	43	(35–50)	66	49	(41–58)	8	20	(10–35)	0.002
Pregnancy assessment	163	94	(89–97)	132	98	(95–100)	31	78	(62–88)	< 0.001
Treatment and dosage										
1st trimester										
Correct drug named	144	83	(76–88)	121	90	(84–94)	23	58	(42–71)	< 0.001
Correct drug and dosage	55	32	(25–39)	48	36	(28–44)	7	18	(9–32)	0.05
2nd /3rd Trimester										
Correct drug named	141	81	(75–86)	113	84	(77–90)	28	70	(55–82)	0.07
Correct drug and dosage	60	34	(28–42)	48	36	(28–44)	12	30	(18–45)	0.62
Severe MiP										
Correct drug named	150	86	(80–91)	126	94	(87–97)	24	60	(45–74)	< 0.001
Correct drug and dosage	90	52	(44–59)	73	54	(46–63)	17	43	(29–58)	0.25
Adequate knowledge**										
Correct drug and dosage	19	11	(7–16)	14	10	(6–17)	5	12	(5–26)	0.77
Parasitological diagnosis and correct drug with dosage	18	11	(7–16)	13	10	(6–16)	5	12	(5–26)	0.57

*Fisher’s exact test used for strata with < 5 observations. Acronyms: MTGs- malaria treatment guidelines; MiP - malaria in pregnancy; Tri - trimester of pregnancy

**correct drug and dosage for uncomplicated malaria in all trimesters and complicated malaria

**Table 4:** Provider knowledge of national malaria treatment guidelines in pregnancy comparing respondents from health facilities involved in MFT or MiMBa projects and those not involved

	Overall			Participating in MFT or MiMBa projects			Not Participating in MFT or MiMBa projects			p-value*
	n = 134	%	95% CI	n = 97	%	95% CI	n = 37	%	95% CI	
Awareness of MTGs	83	62	(53–70)	65	67	(57–76)	18	49	(33–64)	0.08
Training in MiP	66	49	(41–58)	54	56	(46–65)	12	32	(20–48)	0.03
Pregnancy assessment	132	99	(95–100)	96	99	(94–100)	36	97	(86–100)	0.48
Treatment and dosage										
1st trimester										
Correct drug named	121	90	(84–94)	92	95	(88–98)	29	78	(63–89)	0.01
Correct drug and dosage	48	36	(28–44)	35	36	(27–46)	13	35	(22–51)	1.0
2nd /3rd Trimester										
Correct drug named	113	84	(77–90)	89	92	(85–96)	24	65	(49–78)	< 0.001
Correct drug and dosage	48	36	(28–44)	32	33	(24–43)	16	43	(29–59)	0.37
Severe MiP										
Correct drug named	126	94	(89–97)	91	94	(87–97)	35	95	(82–99)	1.0
Correct drug and dosage	73	54	(46–63)	53	55	(45–64)	20	54	(38–69)	1.0
Adequate knowledge**										
Correct drug and dosage	14	10	(6–17)	10	10	(6–18)	4	11	(4–25)	1.0
Parasitological diagnosis and correct drug with dosage	13	10	(6–16)	9	9	(5–17)	4	11	(4–25)	0.74

*Fisher exact used for strata with < 5 observations. Acronyms: MFT, multiple first-line therapies, MTG, malaria treatment guidelines, MiP, malaria in pregnancy, Tri, trimester of pregnancy

**correct drug and dosage for uncomplicated malaria in all trimesters and complicated malaria

**Table 5:** Provider access to malaria guidelines, job aids and training in malaria by facility type and participation in MFT and/or MiMBa study

	Overall		Health facilities		Drug outlets		Working in health facilities participating in MFT* and/or MiMBa	
	N = 174	%	N = 134	%	N = 40	%	N = 97	%
Have read the malaria treatment guidelines	99	57	83	62	16	40	65	67
Have a copy of the malaria treatment guidelines	73	42	66	49	7	17	50	52
Have a job aid for malaria case management	88	51	78	58	10	25	67	69
Participated in an initiative by the government to disseminate guidelines	88	51	85	64	3	7	65	67
Training on malaria diagnosis/management	106	61	93	69	13	32	70	72
Training on malaria in pregnancy	74	43	66	49	8	20	54	56
When was training on malaria in pregnancy conducted?	(N = 74)	%	(N = 66)	%	(N = 8)	%	(N = 54)	%
In the last one year	45	61	41	62	4	50	37	69
More than a year	29	39	25	38	4	50	17	31

*MFT- Multiple first-line therapies

### Pregnancy assessment and malaria testing practice in women of childbearing age

Regardless of facility type, most providers reported assessing for pregnancy in women of childbearing age who sought malaria treatment. Almost all those from health facilities (98%) compared to 78% from drug outlets reported assessing for pregnancy (p < 0.001) (Table 3). Malaria diagnosis was reported by 94% (126/134) of health facility providers compared to 83% (33/40) of drug outlet providers. Most health facility providers (59%, 74/126) reported using microscopy for diagnosis, while 85% (28/40) of drug outlet providers used rapid diagnostic tests. More providers from drug outlets (73%) than health facilities (36%) reported treating adults for malaria presumptively, i.e., without a test result (Table 6).

**Table 6:** Provider reported practices in pregnancy testing and diagnosis of malaria by facility type and participation in MFT and/or MiMBa study

	Overall		Health facilities		Drug outlets		Working in health facilities participating in MFT* and/or MiMBa	
	N = 174	%	N = 134	%	N = 40	%	N = 97	%
Assess for pregnancy in a woman of childbearing age seeking treatment for malaria								
Always	138	79	121	90	17	43	90	93
Sometimes	25	14	11	8	14	35	6	6
Never	11	6	2	2	9	22	1	1
Insist on laboratory malaria diagnosis	159	91	126	94	33	83	90	93
What type of test?								
Blood smear/microscopy	79	50	74	59	5	15	50	52
RDT	80	50	52	41	28	85	40	41
Presumptive clinical malaria treatment in adults								
Always	6	3	2	2	4	10	1	1
Sometimes	71	41	46	34	25	63	29	30
Never	97	56	86	64	11	27	67	69

*MFT- Multiple first-line therapies

### Knowledge of anti-malarial drugs used in pregnancy

Overall, 83% of providers correctly noted that quinine and clindamycin, or artemether-lumefantrine if quinine is unavailable, should be used to treat uncomplicated malaria in the first trimester. Among those reporting use of other drugs, 19% (14/174) mentioned sulfadoxine-pyrimethamine, which is not considered suitable for the treatment of uncomplicated malaria and is not recommended in the first trimester, 4% (7/174) artesunate or artemether and 0.6% (1/174) amodiaquine. More providers from health facilities than drug outlets could correctly name the drug recommended for the treatment of uncomplicated malaria in the first trimester (90% vs. 58%, p < 0.001), second and third trimester (84% vs. 70%, p = 0.07), and severe malaria (94% vs. 60%, p < 0.001) (Table 3). However, few could give the correct dosages, with no significant differences between providers from health facilities and drug outlets; only 10% of providers from health facilities and 12% from drug outlets had adequate knowledge of malaria treatment in pregnancy (p = 0.77) (Table 3).

More providers from health facilities in the MFT or MiMBa projects compared to non-project facilities could correctly name the drug for malaria treatment in the first trimester (95% vs78%, p = 0.01), second and third trimesters (92% vs. 65%, p < 0.001). No difference was seen in the knowledge of treatment drugs for severe malaria (94% vs. 95%, p = 1.0) (Table 4). Providers from facilities in the MFT and MiMBa projects did not demonstrate better knowledge of drug dosages for malaria in pregnancy than those from non-project health facilities. Only 10% of providers from health facilities in the MFT and MiMBa projects had adequate knowledge of malaria treatment compared to 11% of non-participating health facilities (p = 1.0) (Table 4).

### Predictors of adequate healthcare provider knowledge on treatment of malaria in pregnancy

Healthcare providers were considered to have adequate knowledge if they could correctly name the recommended drug and dose for treatment of uncomplicated and severe malaria in each trimester. Facility type (public vs. private) and having received training in malaria in pregnancy were not found to be associated with adequate provider knowledge of drugs and dosages for malaria in pregnancy. Other factors such as age, gender, respondent cadre, professional qualification, level and level of facility (health centre, dispensary, hospital, registered pharmacy, or non-pharmacy drug store), and managing authority were also not significantly associated with better provider knowledge. No differences were noted between the crude and adjusted odds (Table 7).

**Table 7:** Potential predictors of adequate knowledge of malaria in pregnancy treatment

Provider characteristic	N	%	Crude OR	95% CI	p-value	Adjusted OR	95% CI	p-value
Facility classification (reference: Health facilities)	174	100						
Drug outlets	40	23	1.02	(0.9–1.1)	0.72	1.02	(0.9–1.2)	0.69
Training in malaria in pregnancy (reference = No)	174	100						
Yes	74	43	1.04	(0.9–1.1)	0.35	1.04	(0.9–1.1)	0.47
Age (Years)	174	100	1.00	(0.9-1.0)	0.19	1.00	(0.9-1.0)	0.26
Sex (reference: male)	174	100	0.97	(0.9–1.1)	0.5	0.97	(0.9–1.1)	0.59
Participation in MFT or MiMBa (reference: Yes) (only Health facilities)	134	100						
No	37	28	1.0	(0.9–1.1)	0.93	–	–	–
Health Facility type (reference: Public)	134	100						
Private facilities	62	46	0.99	(0.9–1.1)	0.79	–	–	–

*MFT- Multiple first-line therapies

## Discussion

The study aimed to assess knowledge and adherence to malaria in pregnancy case management guidelines in WOCBA among healthcare providers in the context of the Kenyan Ministry of Health MFT pilot in Western Kenya. Providers from health facilities were more aware of national malaria treatment guidelines, more likely to have been recipients of government initiatives that disseminated guidelines, more likely to have a job aid, more likely assessed for pregnancy in WOCBA and had more knowledge about the drugs for the treatment of malaria in pregnancy than those from drug outlets. Although providers from health facilities were more likely to have been trained on malaria in pregnancy case management than those from drug outlets, less than half of providers from health facilities and almost none from drug outlets had received any training on malaria management in pregnancy. There was poor knowledge of anti-malarial drug dosages amongst all providers.

Findings that health facility providers were more aware of malaria guidelines and more likely to have been trained than those in drug outlets have been reported in a 2012 Kenyan study, where only 16% of providers had received training on using AL for malaria treatment, leading to the recommendation that guideline changes be accompanied by activities involving all sector players in unbiased strategies [16]. The findings of the present study concur that drug outlet providers be considered in training, guideline dissemination activities, and supply of wall charts and other job aids. Strategies targeting drug outlet providers have been found to have the potential to improve malaria case management [30]. Despite the MFT pilot and the training reportedly delivered, almost half of all health facility providers had not received training on malaria in pregnancy case management.

While no training on case management was provided as part of the MiMBa observational study, the project may have increased awareness about drug safety. During the roll-out of the MFT project, training was limited to facility-in-charges from health centres and dispensaries in addition to pharmacists from district and referral hospitals. It was expected that the trained personnel would sensitize other healthcare workers in their facilities. However, health worker strikes in public health facilities during the MFT roll-out may have hampered further sensitization activities. In expanding MFT to other malaria-endemic areas in Kenya or other sub-Saharan African countries, consideration should be given to adequate sensitization of healthcare providers and provision of job aids. While more providers from health facilities involved in the MFT pilot reported being trained in the year preceding the study, their knowledge of anti-malarial drug dosage in pregnancy was no better than those from non-pilot facilities. This points to a need for an audit of the structure and rigour of training offered. Training sessions at the healthcare providers’ place of work, with modules on group problem-solving activities and testimonials, have been reported to be effective in changing healthcare provider practice [31–33]. In addition, programmes that rely solely on in-service training are reported to benefit from periodic refresher training [34].

While most health facility providers reported routinely assessing all WOCBA for pregnancy, about a quarter from drug outlets did not. In the qualitative component of the study (Pers. commun, Osoro, 2023), a different group of drug outlet providers reported a lack of pregnancy detection tests which may explain the findings. A few health facility and drug outlet providers reported relying on clinical malaria diagnosis. This has been reported in several meta-analyses where lower compliance to negative malaria RDT results and presumptive malaria treatment were reported [14, 35]. Healthcare providers need to be sensitized to the fact that adherence to RDT test results does not result in worse clinical outcomes compared to presumptive treatment [36].

Providers interviewed for the qualitative component of the study reported frequent stockouts of RDTs in health facilities and drug outlets (Pers. commun, Osoro, 2023). This may explain some of the practices of clinical diagnosis of malaria. Health facility providers reported using microscopy more than RDTs for malaria diagnosis. Possible explanations for this are a preference for microscopy due to perceived higher sensitivity over RDTs in health facilities where laboratories and trained laboratory staff are available, as also expressed by providers in the qualitative component of the study and previous studies in Kenya (Pers. commun, Osoro, 2023) [37, 38].

The lack of knowledge on anti-malarial drug dosages for pregnancy noted in health facilities and drug outlets is not unique to this study. Prior studies in Kenya and other countries have reported the same [14, 20, 39–42]. This study revealed that providers from health facilities in the MFT pilot had better knowledge of the name of the drug but not the dosage. This could be attributed to inadequate training during the MFT roll-out. In the qualitative component of the study, healthcare providers expressed confusion on how to use the anti-malarial drugs in the MFT project and they were unsure which drugs were safe for pregnancy (Pers. commun, Osoro, 2023). From the study findings, no healthcare provider mentioned any new ACT used in the MFT pilot as a treatment for malaria in pregnancy. It is noteworthy that providers from health facilities had better knowledge of drugs and dosages for treating severe malaria than those from drug outlets, perhaps because severely ill malaria patients would most likely visit a health facility. While knowledge of anti-malarial drug names is good, poor knowledge of dosages or number of tablets to be prescribed is a concern as it could lead to medication errors.

The study found that almost half of the providers in health facilities and drug outlets had spent a year or less in their job position, pointing to a high staff turnover rate. There is a possibility that staff trained during the roll-out of the MFT pilot had moved on from their positions. The findings stress an urgent need to conduct regular on-job training for all providers and support through job aids and helplines. Furthermore, including current malaria in pregnancy case management guidelines in the health worker pre-service training curricula would be beneficial. Poor knowledge of anti-malarial drugs by drug outlet providers, with some reporting use of sulfadoxine-pyrimethamine for treatment of malaria, reflects an overall lack of awareness of malaria treatment, particularly in the first trimester. With more than 40% of antimalarials reported to be distributed by unregistered pharmacies in Kenya, adequate training in malaria case management for drug outlet providers is imperative [43]. While most providers in the study held relevant clinical degrees, some had only primary or secondary education. This is of significant concern, as these personnel prescribed drugs to pregnant patients. Frequent supervision of health facilities, in addition to in-service malaria training, and provision of guidelines and job aids, has been shown to significantly improve the quality of malaria treatment practices [44]. The combination of in-service training and supervision has been reported to be more effective in improving healthcare provider practice than using either strategy alone [33]. The need for regular and strict supervision of healthcare providers has been previously called for in Kenya [44]. The study findings suggest that supervision should place greater emphasis on drug outlets where there are more unqualified providers.

Strengths of this study are the inclusion of public, private, and faith-based health facilities and drug outlet providers and the fact that this was part of a mixed-methods study where qualitative findings were used to explain some of the results. Limitations are that healthcare provider knowledge on malaria management in pregnancy was self-reported and pregnant women were not interviewed, so provider practice was not verified nor triangulated with women’s perspectives. In addition, facilities with more than three months of anti-malarial drug stockouts were excluded; thus, the results are likely to have underestimated the extent of the lack of knowledge among healthcare providers in the study area.

## Conclusion

Overall, healthcare providers working in health facilities had better knowledge of case management of malaria in pregnancy than those working in drug outlets. Knowledge of anti-malarial drug dosages among providers in the MFT pilot was no better than those from non-MFT facilities. Alongside the roll-out of the MFT pilot, the National Malaria Control Programme needs to prioritize robust training programmes, supervision, and regulation of drug outlets and health facilities while increasing awareness of the safety of anti-malarials in pregnancy, particularly given the recent change in recommendation for artemether-lumefantrine use in the first trimester.

### Supplementary Information

**Additional file 1.** The Strengthening the reporting of observational studies in epidemiology (STROBE) checklist.

## Data Availability

The study data are available on reasonable request. Interested researchers should contact the corresponding author on the email provided.

## Abbreviations

ACT: Artemisinin-based combination therapy
AL: Artemether-lumefantrine
CDC: Centers for Disease Control and Prevention
CHVs: Community health volunteers
DO: Drug Outlets
HF: Health Facilities
MFT: Multiple first-line therapies
RDTs: Rapid diagnostic tests
PA: Pyronaridine-artesunate
WHO: World Health Organization
WHO-AFRO: WHO- Africa Region
WOCBA: Women-of-childbearing-age
